# Editorial: Biomaterials for chronic wound healing

**DOI:** 10.3389/fbioe.2024.1430646

**Published:** 2024-05-30

**Authors:** Olga Kammona, Maria Vamvakaki, Candan Tamerler

**Affiliations:** ^1^ Centre for Research and Technology Hellas (CERTH), Chemical Process and Energy Resources Research Institute, Thessaloniki, Greece; ^2^ Department of Materials Science and Engineering, University of Crete, Heraklion, Greece; ^3^ Institute of Electronic Structure and Laser, Foundation for Research and Technology—Hellas, Heraklion, Greece; ^4^ Bioengineering Program, University of Kansas, Lawrence, KS, United States; ^5^ Institute for Bioengineering Research, University of Kansas, Lawrence, KS, United States; ^6^ Department of Mechanical Engineering, University of Kansas, Lawrence, KS, United States

**Keywords:** biomaterials, wound healing, hydrogels, wound dressings, antibacterial properties

Wound healing is a multi-stage process during which the injured tissue is repaired and its integrity is restored. This dynamic, interactive process encompasses soluble mediators, various cell types such as parenchymal cells and blood cells, and their associated extracellular matrix (ECM). It also comprises a series of physiological and biochemical events following four distinct, occasionally overlapping healing stages: hemostasis, inflammation, proliferation, and remodeling. A wound is considered chronic if it does not heal due to a physiological impairment of the healing cycle, and remain at the inflammatory stage. Non-healing chronic wounds can be as a result of local and systemic factors including underlying diseases such as diabetic, pressure, arterial and venous ulcers. Chronic wounds are one of the greatest healthcare concerns due to prolonged hospitalization and reduced quality of life and remains a major and persistent problem for dermatology. They are characterized by an elevated and prolonged inflammatory stage, leading to the damage of the ECM and in turn affecting resident fibroblasts. It includes alteration of fibroblast abilities to proliferate and to synthesize and remodel the ECM as the fibroblasts associated with the wound develop a senescent phenotype. Finally, the environment of the chronic wound impairs angiogenesis resulting in chronic hypoxia and stalls epithelialization. The presence of various pathological conditions could further adversely affect wound healing and lead to numerous complications. Since several factors may impair the healing process including diverse range of local and systemic factors, the resultant delayed or impaired wound healing has become a key therapeutic challenge.

The chronic wound treatment often includes debridement, cleaning of the wound and wound dressing. When a trauma occurs, the injured skin should be instantly covered with a wound dressing which could maintain a moderately moist environment for skin regeneration, remove excessive exudates, alleviate pain and prevent infection. The dressing material should preferably satisfy the following requirements: softness and flexibility, high mechanical strength and elasticity, physical and chemical stability, biocompatibility/biodegradability, antibacterial and anti-inflammatory properties and facile applicability. Additionally, wound dressings should be effortlessly sterilized, be cost-effective and have a long shelf life. Existing commercially available wound dressings can only meet a few of the aforementioned specifications. Therefore, novel frontiers for wound dressings should include multi-functional antimicrobial strategies capable of overcoming the present challenges, providing critical cellular components to enhance angiogenesis, and promote wound healing. These should be appropriate for treating impaired and difficult-to-heal wounds, such as diabetic ulcers, and ensure faster healing via reduction of infection, prevention of hypoxia, stimulation of the healing mechanisms, speed up of wound closure, and reduction of scar formation.

In this respect, Frontiers in Bioengineering and Biotechnology (Section Biomaterials) developed a Research Topic titled “*Biomaterials for Chronic Wound Healing*” which was co-edited by M. Vamvakaki, C. Tamerler and O. Kammona (2024). Nine papers were submitted to this Research Topic, of which four were accepted and published (Reviews, N = 3; Original Research, N = 1).

Advancements in materials science and tissue engineering led to the design and production of novel wound dressings based on natural or synthetic biomaterials and/or their combinations, aiming to accelerate the wound healing process. These novel dressings are characterized by biocompatibility/biodegradability, minimal toxicity, durability, hydrophilicity, increased absorption/permeability, antimicrobial properties, and enhanced capacity for skin tissue repair. Ansari and Darvishi presented the requirements for an efficient wound dressing and the different stages of wound healing. They subsequently reviewed various natural biomaterials such as silk, fibrin, collagen, hyaluronic acid, gelatin, N-acetylglucosamine with respect to their sources, synthesis processes and usage in wound dressings. Furthermore, they reviewed common synthetic biomaterials and provided their properties including biocompatibility, biodegradability, bioabsorbability, toxicity and covered their synthesis and modifications with different molecules. The authors summarized the quality control techniques and covered *in vitro*, *ex vivo* and *in vivo* quality assays for validations of their effectiveness when produced as wound dressings.

The next Research Topic covered in this issue were based on hydrogels. Hydrogels have a healing effect on wounds due to their moisturizing, cooling and pain-relieving ability. Among them, hydrogels based on gelatin methacrylamide (GelMA) exhibit significant potential for skin tissue engineering owing to their tunable and favorable properties and their similarity to the ECM of skin. GelMA hydrogels can efficiently promote wound healing and skin tissue remodeling via reduction of inflammation, facilitation of vascularization, and support of cell growth. Zhang et al. reviewed the application of GelMA-based hydrogels in wound dressings, skin tissue engineering and transdermal drug delivery. Liang et al. designed and developed GelMA-based wound dressings encapsulating antibacterial polypeptides and MXene nanoparticles (NPs). The composite hydrogels were characterized by enhanced swelling ratio and mechanical strength, antibacterial properties and effective degradation rate. They were also shown to promote cell proliferation and adhesion *in vitro* and to accelerate wound closure, reduce inflammation and speed up epithelial formation and maturation *in vivo*. In conclusion, the newly developed hydrogels were found to exhibit superior tissue regeneration ability.

**FIGURE 1:**
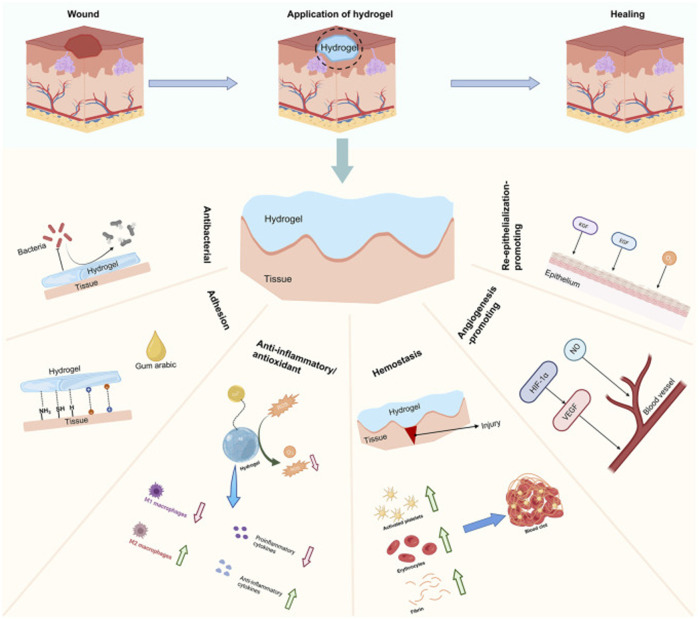
Classification of functional hydrogels (Hao et al.).

The final topic was focused on the stimuli responsive hydrogels. Rapid and efficient healing of maxillofacial and oral trauma is a major concern to both clinicians and patients. Functional hydrogels capable of delivering various cargos that promote wound healing, such as small-molecule drugs, cytokines, nucleic acids, exosomes, stem cells and nanomaterials, have been found to have a positive effect on the healing of maxillofacial and oral injuries. Apart from targeted and controlled release of active agents, multifunctional drug-delivery hydrogels play an active role in stimulating wound healing and have demonstrated unique performance in wound dressings via their adhesive, hemostatic, antioxidant, anti-inflammatory, angiogenic and antibacterial properties, their ability to promote re-epithelialization and effectively seal wounds (Figure 1). Additionally, functional hydrogels are capable of responding to variations in temperature, pH, light, reactive oxygen species and light to release active agents, thus enabling accurate treatment. In this respect, Hao et al. described the structure of the oral mucosal and its healing process, and summarized the presently available stimuli-responsive hydrogels that are used as wound healing promoters.

